# Notes and News

**Published:** 1903-05-02

**Authors:** 


### Notes and News.

The Military Hospital at Alton, Hants, is to be opened on May 1st by the Princess Louise.

There is a good deal of small-pox in Dublin, imported, it is said, from Liverpool.

Cigarettes will be at a premium amongst the youth of Pennsylvania, where a Bill has been passed forbidding the sale of cigarettes to persons under 21 years of age.

Sir William Crookes and Professor Dewar, commenting upon the quality of the London water supply in March, say "the general condition of the water supply was never more carefully superintended or more satisfactory."

A controversy is proceeding in the pages of the *Westminster Review* between Dr. Knott and Mr. G. Greenwood relative to the medical knowledge displayed by Shakespeare, bearing more particularly upon Harvey's discovery of the circulation of the blood. Dr. Knott indicates that this was foreshadowed by Shakespeare, and that this contention is borne out in his writings. Mr. Greenwood disputes both the interpretation and quotations made by Dr. Knott.

An inquiry into the food inspection of the Port of London has been instituted. The object of the inquiry is to ascertain whether it is desirable to extend the attributes of the Port Sanitary Authority in order that the one authority might inspect food elsewhere. One witness at the first sitting of the inquiry stated that if the rigorous inspection of the Port of London Sanitary Authority were not extended to wharves outside the docks, people would take their goods elsewhere.

Mr. George A. Arnaudin (late assistant secretary to the Farningham and Swanley Homes for Little Boys) has been appointed superintendent of St. John's Hospital for Diseases of the Skin, Leicester Square.

The Orient steamer *Orizaba* called at Plymouth from Sydney on Monday and reported that one of the crew was landed at Suez suffering from the plague. The Plymouth authorities demanded that she should be treated as an infected ship.

The hoped-for banishment of plague from Cape Colony and Natal has not been attained. Rats appear to be responsible for a rather widespread recrudescence, originating, as far as can be ascertained, from a ship arriving from India.

The Society of National Refuges for Homeless and Destitute Children, which held its annual meeting on Tuesday, is doing an admirable work in providing useful and healthy work for hundreds of boys and girls. They secure excellent opportunities for boys to enter the Navy and merchant service, after training on the ships *Arethusa* and *Chichester*. The children come from all parts of the country, and therefore the society has a wide claim to support.

The Lord Mayor has consented to assist the arbitration between St. Luke's Hospital for Lunatics and the landlords of St. Bartholomew's Hospital. It is contended that as St. Luke's needs for its own sake to move from its present site, that it ought to vacate and not claim compensation for buildings. The administration of St. Luke's hold a different view, hence the appeal to arbitration.

The chairman of the Soldiers' and Sailors' Homes Fund is appealing to the public to devote the penny in the pound which represents the abatement in income-tax over that generally expected, to furthering the scheme to provide 27 homes for disabled Army and Navy men. Any scheme to assist our soldiers and sailors is bound to be popular, but we have already pointed out that the almshouse system is open to so many objections that it is doubtful whether the homes will be as much appreciated as the kind-hearted public hope and expect.

Portraits of great men will always have something more than a passing interest for those who come after them, and especially so if they have been drawn by an artist who had the advantage of being well acquainted with the peculiar characteristics of his sitter. It may be well, therefore, to mention that a very good plate has been issued of the well-known picture of Sir Richard Owen by Thaddeus. In this portrait, which was taken in later life, the professor is shown in academic costume, and in a characteristic attitude, discoursing upon the skull of some prehistoric monster such as his heart loved. The bright eyes and expression of profound thought that were striking features of his physiognomy are well brought out; in fact, the picture possesses that subtle psychological factor that is essential to a good portrait. The excellent engraving is published by Paul Naumann, Pentonville Road, N.

